# Reducing Unspecific Protein Adsorption in Microfluidic Papers Using Fiber-Attached Polymer Hydrogels

**DOI:** 10.3390/s21196348

**Published:** 2021-09-23

**Authors:** Alexander Ritter von Stockert, Anna Luongo, Markus Langhans, Thomas Brandstetter, Jürgen Rühe, Tobias Meckel, Markus Biesalski

**Affiliations:** 1 Laboratory of Macromolecular Chemistry and Paper Chemistry (MAP), Department of Chemistry, Technische Universität Darmstadt, 64287 Darmstadt, Germany; alexander.von_stockert@tu-darmstadt.de (A.R.v.S.); markus.langhans@tu-darmstadt.de (M.L.); tobias.meckel@tu-darmstadt.de (T.M.); 2 Laboratory of Chemistry and Physics of Interfaces, Institute for Microsystems Technology, Technical Faculty, University of Freiburg, 79110 Freiburg, Germany; anna.luongo@imtek.uni-freiburg.de (A.L.); brandstetter@imtek.uni-freiburg.de (T.B.)

**Keywords:** POC, µPAD, entropic shielding, cellulose, microfluidics, protein binding, surface functionalization

## Abstract

Microfluidic paper combines pump-free water transport at low cost with a high degree of sustainability, as well as good availability of the paper-forming cellulosic material, thus making it an attractive candidate for point-of-care (POC) analytics and diagnostics. Although a number of interesting demonstrators for such paper devices have been reported to date, a number of challenges still exist, which limit a successful transfer into marketable applications. A strong limitation in this respect is the (unspecific) adsorption of protein analytes to the paper fibers during the lateral flow assay. This interaction may significantly reduce the amount of analyte that reaches the detection zone of the microfluidic paper-based analytical device (µPAD), thereby reducing its overall sensitivity. Here, we introduce a novel approach on reducing the nonspecific adsorption of proteins to lab-made paper sheets for the use in µPADs. To this, cotton linter fibers in lab-formed additive-free paper sheets are modified with a surrounding thin hydrogel layer generated from photo-crosslinked, benzophenone functionalized copolymers based on poly-(oligo-ethylene glycol methacrylate) (POEGMA) and poly-dimethyl acrylamide (PDMAA). This, as we show in tests similar to lateral flow assays, significantly reduces unspecific binding of model proteins. Furthermore, by evaporating the transport fluid during the microfluidic run at the end of the paper strip through local heating, model proteins can almost quantitatively be accumulated in that zone. The possibility of complete, almost quantitative protein transport in a µPAD opens up new opportunities to significantly improve the signal-to-noise (S/N) ratio of paper-based lateral flow assays.

## 1. Introduction

The ASSURED requirements are given by the World Health Organization (WHO) as a benchmark for POC diagnostics [1]. ASSURED stands for affordable, sensitive, specific, user-friendly, rapid and robust, equipment-free, environmentally friendly, and deliverable to end-users. In a recent study, the term ASSURED has been changed to REASSURED, proposing the inclusion of real-time connectivity to smart phones for superior read-out and decision making, as well as ease of specimen collection [2]. Cellulose is a very inexpensive, sustainable, and chemically stable starting material, and papermaking is a well-established process, making potential POC µPADs a reasonable candidate for (RE)ASSURED diagnostics [1,3,4,5,6,7,8,9]. Dipstick and lateral flow tests have been known for more than 60 years and were originally designed for clinical diagnostics [10]. Due to their quick and easy use, these tests have proven themselves in a lot of different fields, such as agriculture, environment surveillance, food safety, and even drug delivery [8,11,12,13,14]. It is reasonable to assume that the use of such rapid tests will continue to increase and that further areas of application will be opened up to replace expensive and time-consuming laboratory diagnostics. This makes it all the more important to strive to produce these tests entirely from renewable materials [15,16,17]. Most of the properties of the materials used in today’s lateral flow tests, such as glass fiber meshes, plastic foils, or nitrocellulose membranes, respectively, can be replaced by paper with relatively little effort. Capillary transport in paper can be fine-tuned in many ways [3,18,19], protein binding can be achieved to high capacities [20], and the use of paper eliminates the need for (typical plastic) backings, as with commercially available lateral flow tests (LFTs) [15,17,21,22]. We believe that paper-only lateral flow tests have the potential to perform similarly to alternatives, such as nitrocellulose membranes and assembled lateral flow assays, and, at the same time, account for a much higher degree of sustainability.

One general limitation of LFTs is their low sensitivity (i.e., S/N ratio) when compared to tests that are carried out in an analytical laboratory [9,17,23]. This low S/N ratio can result from multiple sources, such as light scattering in the read-out zone or the retention of analyte across the length of the assay. The low S/N ratio on paper- or nitrocellulose-based analytical devices limits their use to tests, where enough analyte is present to result in a visible signal at the test line. As the amount of analyte is a limiting factor for these tests, nitrocellulose (NC)-based assays cover the surface of the NC membrane with bovine serum albumin (BSA), which is also known as “blocking”, in order to reduce unspecific protein binding [24,25]. The bound protein layer leads to reduced binding of the analyte during the actual LFT and, therefore, maximizes the amount of detectable analyte at the test zone. This technique works well, as proteins have a very high affinity to bare nitrocellulose. Whether this well-established surface-shielding approach is suitable for paper-based assays has not yet been studied in detail. In particular, very little is known to date about whether the affinity of proteins, such as BSA to cellulose, is high enough to remain bound through the course of an assay performed in paper-only LFTs. A general issue of using BSA in the context of paper-based assays is linked to the fact that paper fibers swell in aqueous environments. Thereby, a change in surface coverage of BSA on the fiber may easily occur due to the fact that these proteins are only physically attached to the cellulosic materials [26,27].

Instead of using BSA protein blocking to prevent unspecific binding of an analyte to cellulose, one may also consider other shielding layers to modify paper fiber surfaces in order to maximize the detectable analyte. Prominent examples are, e.g., polymeric hydrogels. With respect to such shielding layers on model surfaces (mostly planar, solid surfaces), many successful examples use surface-attached hydrogels that significantly reduce the binding of proteins to surfaces due to an entropic shielding effect [28,29,30]. The key idea here is that polymer and proteins have no enthalpic interactions and a penetration of the protein would cause an additional stretching of the surface-attached polymer layer and, thus, a large loss of entropy. Additionally, size exclusion effect might contribute to the protein exclusion. A protein, as a potential analyte, is now unable to penetrate the hydrogel layer. Theoretically, the protein should, therefore, be unable to interact with the coated surface and flow above the hydrogel layer. One example that exhibits such a shielding effect once bound as a thin layer to a surface is polyethylene glycol (PEG), which has been used in medicine for over 20 years [31,32]. PEG and other hydrogels, such as PDMAA, have been used on organic and inorganic surfaces and were shown to reduce protein adhesion by orders of magnitude [33,34,35].

Whereas the shielding behavior of surface-attached polymer hydrogels has been investigated in many different studies, paper fibers have been, so far, rarely considered in this context. The latter may directly be related to the more complex geometrical and chemical structure of the paper-constituting fibers. These fibers are often described as hollow lignocellulosic tubes with a certain wall thickness. This description, however, barely begins to describe the complex structures that this plant material has. Paper fibers come in many shapes and forms, even when only focusing on a single fiber type. The fiber length, thickness, wall thickness, kinks, and curls are some of the many parameters that are different for each fiber in each fiber type [36]. Many of these fiber-specific characteristics have a direct influence on the fluid dynamics inside a paper channel and are, therefore, interesting candidates for fine-tuning the capillary flow [37]. Meanwhile, other groups largely focus on paper extrinsic manipulation of microfluidics [38,39].

In a study by Hoare et al. [40], Whatman 40 paper sheets were coated in a two-step mechanism with an oligoethylene-glycol-based polymer that is able to form a hydrogel layer. In a first step, an aldehyde-functional copolymer was thermally bound to the paper fiber surface, with a consecutive binding of another OEGMA copolymer carrying hydrazine groups that react with the aldehyde moieties accessible from the first polymer coat. Using such POEGMA-coated paper sheets, the authors discovered that this coating was able to increase the read-out of β-galactosidase on their coated paper strips when compared to uncoated or BSA-blocked papers. This study already proves that hydrogel-coated papers have high potential for further POC diagnostics; however, a detailed analysis of the protein distribution across the paper strips was not conducted. Additionally, the use of filter paper may have an effect on the interactions between fiber and analyte, as filter papers, while not containing any fillers, such as CaCO_3_, still contain chemical additives, such as wet strength agents.

As an alternative to the aforementioned thermal binding of hydrogels, polymer layers can also be attached to paper fibers by a photochemical bonding step in a very simple and efficient approach [2,8,26]. In brief, using a photoreactive group, such as 4-methacryloyloxy benzophenone(MABP), as a photo-crosslinking unit embedded in a pre-formed polymer enables the modification of surfaces carrying aliphatic CH groups, e.g., organic substances, including cellulose (i.e., paper fibers). As the photoreactive polymer itself contains CH groups, inter- and intramolecular crosslinking within the polymer is also present, creating a crosslinked hydrogel on such organic surfaces. A hydrophilic copolymer used in many of our studies is PDMAA, which, if crosslinked by UV light, creates a surface-linked hydrogel. In different studies, this hydrogel has already proven a very useful shielding layer to prevent protein adsorption on planar glass or plastic surfaces [29,41]. In addition, binding of PDMAA to paper fibers has also been shown to increase paper wet strength properties [42]; however, shielding effects in terms of generating a protein-repellent surface coating on paper fibers have not yet been studied.

Here, we investigate how paper-fiber-linked polymer hydrogels influence the interaction of protein and modified paper fibers in model studies of paper-based lateral flow devices. As the paper substrates, lab-made cotton linter paper was produced and pre-formed paper sheets were modified with polymer hydrogels using the above-described photochemical approach. The chemical composition of the fiber-attached polymers consists of PEG analogous, methacrylate-based copolymer P(OEGMA-co-MABP), as well as a P(DMAA-co-MABP) copolymer; by using fluorescent or fluorescently labelled proteins in combination with sensitive fluorescent imaging, we are able to provide a semi-quantitative view on the interaction of model proteins with these fiber-immobilized hydrogels. In particular, we study the influence of the chemistry of the hydrogel, the type of flow, in particular, if the protein is applied on a pre-wetted or dry paper sheet, and the influence of paper-intrinsic parameters, such as the grammage of the sheets. Finally, we investigate whether fiber-retained proteins during flow experiments can be concentrated at the end of the flow test strip by evaporation of the fluid medium.

## 2. Materials and Methods

### 2.1. Synthesis of P(DMAA-co-MABP-co-RhBMA)

A total of 3.96 g (4.11 mL, 38.8 mmol, 97.7 mol%) of destabilized dimethyl acrylamide was added to a Schlenk flask containing 213 mg (0.8 mmol; 2.0 mol%) methacryloyl benzophenone (MABP) and 55.4 mg (0.1 mmol; 0.3 mol%) rhodamine-B–methacrylate (RhBMA) in 40 mL dimethyl formamide (DMF), under nitrogen atmosphere. Afterwards, 20 mg (0.12 mmol) of AIBN was added and the solution was degassed with the freeze–pump–thaw method three times. The reaction was carried out under a nitrogen atmosphere at 60 °C for 16 h and stopped by transferring the solution to 800 mL of diethyl ether. A white precipitate formed, which was isolated by using a centrifuge. The product was then resolved in chloroform and precipitated again from diethyl ether, before being dried in vacuo. The MABP content of the polymer was analyzed with ^1^H-NMR spectroscopy (Figure S1) and determined to be 2.4%. The M_n_ of the polymer was determined to be around 28,000 g/mol.

### 2.2. Synthesis of P(OEGMA-co-MABP-co-RhBMA)

A total of 9.775 g (9.5 mL, 19.55 mmol, 97.8 mol%) of destabilized dimethyl acrylamide was added to a Schlenk flask containing 106 mg (0.4 mmol; 2 mol%) MABP and 27.7 mg (0.05 mmol; 0.25 mol%) RhBMA in 40 mL dimethylformamide (DMF), under nitrogen atmosphere. Lastly, 12 mg (0.8 mmol) of AIBN was added and the solution was degassed with the freeze–pump–thaw method three times. The reaction was carried out under a nitrogen atmosphere at 60 °C for 16 h and stopped by transferring the solution to 800 mL of diethyl ether. A pink gel formed at the bottom of the flask, which was isolated by dissolving it in chloroform. Reprecipitation yielded a pure product that was, again, isolated by dissolution in chloroform, followed by evaporation of the solvent. The MABP content of the polymer was analyzed with ^1^H-NMR spectroscopy (Figure S2) and determined to be 1.7%. The M_n_ was estimated by GPC to be around 500,000 g/mol.

### 2.3. Sheet-Forming of Cotton Linter Paper

The sheets were formed with an HAAGE sheet former BB at varying fiber concentrations in order to achieve different basis weights of 50, 84, and 120 g·m^−2^ within the paper sheets, respectively. The procedure of DIN/ISO norms were followed and the papers dried at 93 °C at reduced pressure for 10 min. The paper sheets were cut into 8.0 cm × 0.5 cm strips and stored in a climate room at 23 °C and 50% relative humidity.

### 2.4. Dip-Coating and Subsequent Photo-Crosslinking of Polymers

The polymers were dissolved in water at 30 mg/mL (PDMAA) and 25 mg/mL (POEGMA), respectively. The substrate was placed into the dip-coating solution for 15 s. Once retrieved, the papers were placed onto a Teflon dish and placed in a photo-chamber equipped with a UV lamp. UV crosslinking was performed with a 1000 W Mercury/Xenon lamp by Newport Corporation, in combination with an I-line filter for specific illumination at 365 nm. The specimen was illuminated once from each side with 16 J·cm^−2^ for each illumination step. After the crosslinking process, excess polymer was removed by extraction with water. The successful crosslinking could then be confirmed with IR spectroscopy.

### 2.5. Flow Experiments

The flow setup was achieved by using two laboratory jacks, one of which held a fluid reserve (3 mL PBS-pH 7.4) and the second of which carried a poly(methyl methacrylate) (PMMA) slide, onto which the paper strip was placed. The strip was positioned in a way that 0.5 cm overlapped on one side of the PMMA slide. This overlap was then placed at the edge of the lab jack in a way that, once the fluid reserve was raised to the same level as the strip, the fluid came into contact with the strip and began to imbibe into the porous material.

Protein application was done in three separate ways. The concentration of the proteins was chosen in a way that the protein was easily visible with fluorescent imaging techniques. Once the concentration was chosen, the protein was always applied in 5 µL solution and always at the 1 cm mark of the strip (0.5 cm from the start of the PMMA slide). The timing of application varied throughout the three different sets of experiments. The “fully wetted flow” experiments were carried out with the protein being applied once the fluid front had approached 3 cm into the strip. This was done to ensure a fully swollen hydrogel on the paper surface. In the “with the front” experiments, the protein was applied with the fluid front once the 1 cm mark was reached on the strip. In the “wet out flow” experiments, the protein droplet was applied before the strip was connected with the fluid reservoir.

### 2.6. (Un)specific Binding of Proteins to Fibers

To determine the specific binding of CBM 3a–mRuby3 to coated and uncoated cellulose, a piece of paper was placed into a protein solution of ~2 µg/mL for 10 min and subsequently washed for 2 min in PBS. A comparison of the fluorescent intensity was determined using confocal microscopy.

### 2.7. Chemicals

Poly(ethylene glycol) methyl ether methacrylate (Mn = 500) was purchased from Aldrich and destabilized over basic alumina. Dimethylacrylamide was purchased from Fisher Scientific and destabilized over basic alumina. Benzophenone 99% was purchased from Aldrich. Rhodamine B was purchased from Sigma. AIBN was purchased from Sigma-Aldrich. DMF 99.8% was purchased from Acros Organics. Chloroform 99.8% was bought from Acros Organics. All salts used in the making of PBS buffer solutions were purchased from Carl Roth. BSA-FITC was purchased from Sigma-Aldrich.

### 2.8. Chemical, Contact Angle and Microscopy Analysis

FTIR spectra were taken with the Spectrum 1 FT-IR spectrometer by Perkin Elmer and analyzed using the “Spectrum” software; NMR spectra were measured with the 300 MHz Avance II NMR Spektrometer by Bruker BioSpin GmbH and analyzed using MestReNova 11.0.3.

Contact angles were measured with an OCA 35 from Dataphysics Instruments and modeled with the Software “SCA software version 4.5.2 Build 1052”.

Fluorescent overview images were taken with the Vilber FUSION FX7 EDGE by Vilber using an illumination wavelength of 480 nm and an emission filter of 505–565 nm; Conflocal microscopy was performed with a Leica TCS SP8, equipped with excitation lasers of 405 nm, 588 nm, 552 nm, and 633 nm. The main objective used was a ×10 magnification objective with a numerical aperture of 0.3.

## 3. Results

### 3.1. Modification of Paper Sheets with Functional Polymers by Lithographic Grafting

In a first step, filler- and additive-free paper sheets with a basis grammage of 50, 84, and 120 g·m^−2^, respectively, were prepared using a Rapid-Koethen sheet former. Cotton linters were chosen as the fiber type, as they are mainly composed of cellulose and contain very low amounts of hemicellulose and lignin, respectively. The fibers were refined prior to paper sheet formation to have a Schopper–Riegler (SR) value of 25, as described in detail elsewhere [43]. All papers produced in this work were characterized with respect to their paper-intrinsic properties (grammage, thickness, etc.) using standard methods, prior to further modification and investigation, as described in the Supplementary Information. Prior to the polymer functionalization, the paper was cut into 8.0 × 0.5 cm pieces. Hydrophilic benzophenone-functionalized polymers were synthesized, as reported earlier [41]. In brief, DMAA and OEGMA monomer was polymerized with 2.0 mol% of MABP monomer, respectively, while adding traces of RhBMA monomer to the polymerizations. The latter is used for photodetection of the polymers after attachment to the fibers. All polymers were analyzed via NMR spectroscopy, as well as GPC (Supplementary Information), to determine the molecular characteristics. As seen in ^1^H-NMR analysis, both polymers contain roughly 2% MABP. The PDMAA-based polymer incorporated 2.4 mol% MABP, while the POEGMA copolymer contains 1.7 mol% MABP. Taking the molecular weight of the polymers into account, the MABP content per polymer chain is estimated to be around 7 for P(DMAA-co-MABP) and 17 for P(OEGMA-co-MABP) [18,41,44].

**Figure 1:**
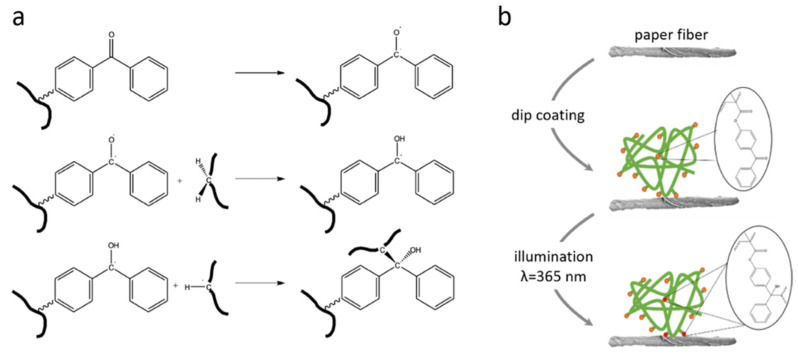
(**a**) Chemical mechanism underlying the crosslinking process. The excited MABP performs a C–H insertion with another molecule via a temporary triplet state. (**b**) Binding of a polymer chain to a cellulose fiber (not to scale), with the orange dots symbolizing unreacted MABP units and the red dots crosslinked MABP.

The process of polymer modification of the paper sheets is schematically outlined in Figure 1. In brief, the PDMAA-based copolymer was dissolved in water at a concentration of 30 mg/mL. This concentration had been determined earlier to be sufficient for coating paper fibers [43]. POEGMA-based copolymer was used at a concentration of 25 mg/mL in order to stay below the critical value of phase separation. Water was chosen as the solvent of choice, as it swells the fibers [26] and allows for a more homogenous coating compared to coatings generated from ethanol- or butanol-based solutions, as shown in a parallel study (data not shown here) [45]. Dip-coating was chosen as the desired coating process, as the cellulose fibers take up the solution by sorption processes once they come into contact. The capillary action leads to an uptake of roughly 500% of the paper weight in water (depending on the degree of refining of the fibers, Table S1) within a few seconds, rendering dip-coating a fast and effective method for an even coating of polymer throughout the paper sheet. To limit migration of the polymers to the edges of the paper during evaporation, the polymer is crosslinked shortly after dipping using a wavelength of 365 nm and a typical energy dose of 16 J/cm^–2^.

### 3.2. Characterization of Functional Paper

**Figure 2:**
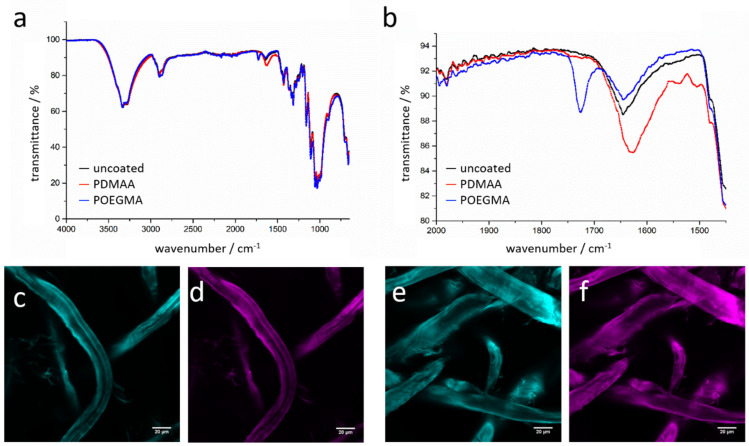
IR spectra of uncoated cotton linter paper (black) in comparison with PDMAA-coated paper (red) and POEGMA-coated paper (blue) are shown (**a**). In a close-up, the changing bands at 1600–1750 cm^–1^ are clearly visible (**b**). CLSM images show cotton linter fibers stained with calcofluor white (**c**,**e**, cyan), as well as the distribution of PDMAA (**d**) and POEGMA (**f**) on the respective fibers (magenta).

FTIR spectroscopy was performed to investigate the chemical identity of the polymer-modified paper (Figure 2). As reference, an unmodified paper sample was measured, and typical absorption bands for the cellulosic nature of the fibers were observed (black line spectrum in Figure 2). Because the polymer-modified paper sheets consist, to a very large extent, of the identical cellulosic material, it is not surprising that the spectra after modification with the respective polymers are dominated by similar absorption bands as compared to the unmodified sheets. However, taking a closer look at distinct absorption bands corresponding to the PDMAA and POEGMA, respectively, reveals important information by a few now observed bands. For the PDMAA-modified paper, an increase at roughly 1630 cm^–1^ can be seen, where the characteristic amide band of this acrylamide species is located. The POEGMA-based copolymer introduces a completely new signal in the FTIR spectrum at 1730 cm^−1^, which can be attributed to the introduction of carbonyl bonds of the ester groups present in this polymer. Hence, FTIR shows that both polymers were present in the investigated samples. The majority of the material, however, remains with the cellulosic fibers. By gravimetric means, we investigated also the increase in mass of the polymer-modified paper samples. Such measurements were performed under normal climate conditions to keep the amount of water sorption into the paper sheet almost constant, expecting a systematic error occurring due to water sorption in varying polymer mass linked to the fibers. Depending on the concentration of the polymer in the dip-coating solution, the amount of fiber-bound polymers can be adjusted between 0 and 12 wt% relative to the fiber mass. As was shown in previous publications, by carefully extracting the polymer-modified paper samples after photo-linking the macromolecules to the fibers, any physically adsorbed polymer chains can be washed out of the paper sheet, leaving only covalently attached polymers inside the paper sheet [18].

We next were interested in inspecting the spatial distribution of the polymers on the paper fibers. We, therefore, used similar copolymers as before; however, we now implemented minute quantities of a fluorescently labeled monomer, rhodamine-B–methacrylate (RhBMA). Paper sheets were modified again by dip-coating with the fluorescent copolymers, and further analyzed by confocal laser scanning microscopy (CLSM). Examples of CLSM images showing PDMAA- and POEGMA-modified paper fibers are depicted in Figure 2d,f, respectively. It can be observed that the fluorescent signal is very homogeneous along the fiber, and no larger areas without any signal are observed. The latter suggests that the polymer is evenly distributed on the fibers, as well as within the cell walls. For all subsequent experiments, the rhodamine-B-free polymer was used.

**Figure 3:**
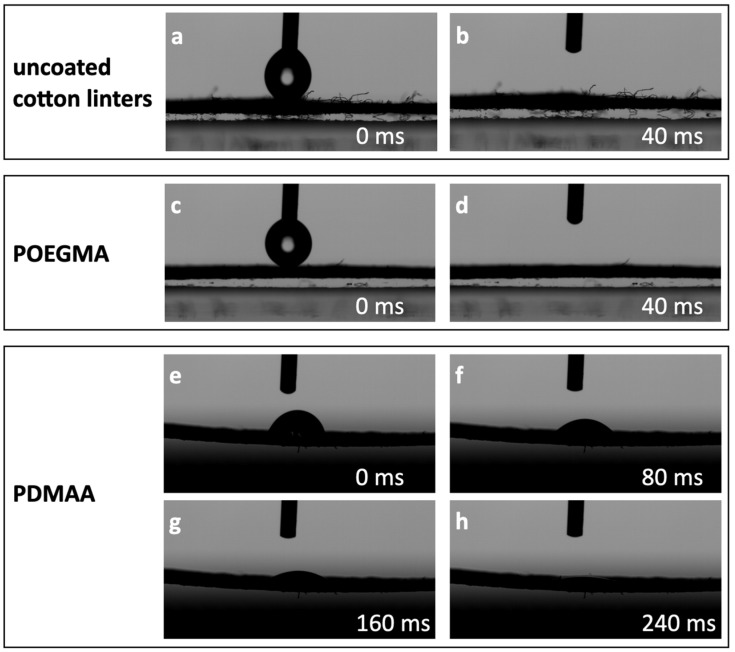
Dynamic water contact angle changes on uncoated (**a**,**b**), POEGMA-coated (**c**,**d**), and PDMAA-coated (**e**–**h**) cotton linter paper. A 2 µL drop of water is applied to the paper, and the time until full imbibition is measured. The total imbibition time for the PDMAA experiment was 7 frames (280 ms), and, for the POEGMA-coated and uncoated paper, less than 1 frame (40 ms).

Since the functionalized papers will be used in the context of protein binding in lateral flow tests, we first tested their interaction with water in imbibition studies. For this, the uptake of a 2 µL drop of water in the untreated and polymer-modified paper sheets was video-captured at a frame rate of 25 Hz. While the water drop imbibes untreated and POEGMA-modified sheets in less than 40 ms, it takes about 280 ms to enter into PDMAA-modified paper (Figure 3). Most likely, the observed difference can be attributed to a change in the contact angle that water forms with the coated surface.

As capillary transport is responsible for the imbibition dynamics and directly proportional to the water contact angle, we investigated the dynamic contact angle of water on model surfaces of P(OEGMA) and P(DMAA-co-MABP), respectively. Both polymers were applied to a PMMA slide via dip-coating and crosslinked once at 16 J/cm^−2^. After subsequent extraction of unbound polymer, the contact angle was measured as a function of time.

**Figure 4:**
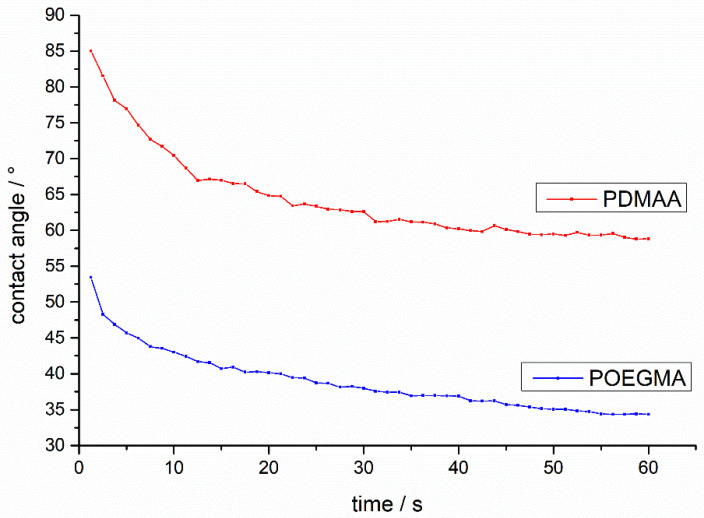
Dynamic contact angle measurements of P(OEGMA-co-MABP) and P(DMAA-co-MABP) on a PMMA slide. The coating was applied via dip-coating, crosslinked, and subsequently extracted for 20 min in water.

The results in Figure 4 show that the PDMAA coating exhibits a much higher contact angle than the POEGMA coating, both immediately after placing the drop on the surface (85° and 55°, respectively), as well after about 60 s, where an almost constant (static) contact angle of about 60° and 35° can be observed. Because the capillary pressure is directly related to the contact angle, a lower contact angle yields faster capillary transport [36]. The latter results suggest that POEGMA-coated paper exhibits a significantly faster transport of water in comparison to PDMAA-coated paper, provided that water–surface interactions on planar substrates with our reference studies behave similarly and the spreading of the water on the polymer-coated fibers is the most important (first) step of the dynamics of imbibition.

### 3.3. Protein Binding Studies under Non-Flow Conditions

**Figure 5:**
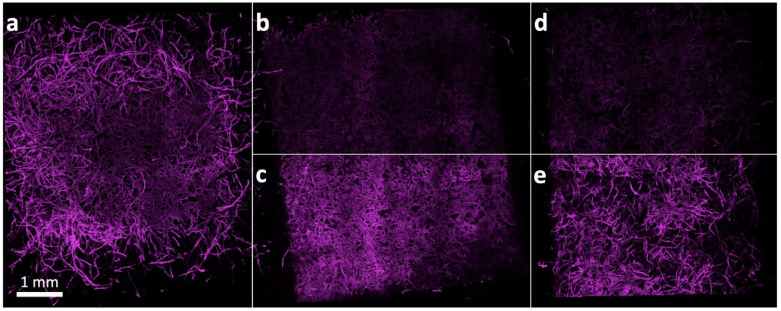
CLSM image of three differently treated cotton linter papers, stained with CBM3a-mRuby3: uncoated (**a**), coated with POEGMA (**b**,**c**), and coated with PDMAA (**d**,**e**). The bottom half of the POEGMA (**c**) and PDMAA (**e**) coated papers is enhanced in brightness for better visualization, while the top half shows the observed intensity in comparison to the uncoated paper.

We next turned our attention towards understanding the interaction of model proteins with our polymer-modified paper sheets in order to investigate a possible protein-shielding effect of the hydrogel fiber coatings. For this, we cloned a fluorescent reporter protein with a high affinity to cellulose. The carbohydrate binding module CBM3a from Clostridium thermocellum that is directed to crystalline cellulose was genetically fused to a fluorescent protein called mRuby3 [46]. By submerging paper in a solution containing ∼2 µg/mL CBM3a–mRuby3, followed by a gentle, two times extraction in phosphate buffered saline (PBS), the remaining fluorescence intensity, i.e., CBM3a–mRuby3 bound to the specimens, was investigated via confocal microscopy. In Figure 5, fluorescence images are shown for an uncoated paper (left) and papers coated with P(DMAA-co-MABP) (center) and P(OEGMA-co-MABP) (right). While the fluorescence image of the uncoated paper is shown in one brightness setting only, the bottom half of the polymer-coated variants is enhanced in brightness for better visualization. The top half of these papers, however, is shown in the same brightness as observed for the uncoated paper, demonstrating that a far lower amount of CBM3a–mRuby3 was able to bind on the polymer-coated papers.

Both the POEGMA- and the PDMAA-coated cellulosic fibers showed a clear reduction in protein retention. This reduction can be attributed to the polymer coating, which blocks the protein from reaching the fibers and binding to them. As CBM3a-mRuby3 has a very high and specific affinity to cellulose, one can further assume that the used model protein did only find very few open spots on the fibers to attach to the cellulosic material and does not bind to the polymer coating itself, i.e., it can be easily washed off the fibers. Note, as is shown by the fluorescent image analysis of the uncoated sample (Figure 5a), the model protein does not come off the fibers, even after rigorous washing. Hence, these results suggest that, with POEGMA- and PDMAA-coated paper sheets, a significant protein-shielding effect exists, which, presumably, could apply to other proteins as well.

### 3.4. Lateral Flow Experiments and Protein Adsorption

**Figure 6:**
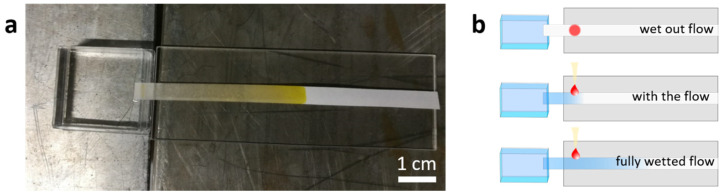
(**a**) Flow experiment with BSA-FITC as the analyte. The paper strip is placed on a PMMA backing and dipped into a fluid reserve, which starts the imbibition process. (**b**) Representation of the three types of protein application. The top image shows the “wet out flow”, the middle image the “with the flow” or “front”, and the bottom image the “fully wetted flow” setup.

In a next step, we investigated the polymer-modified paper sheets in a lateral flow setup. Pre-cut paper strips were placed on a PMAA backing, with a small tip of the paper strip hanging over the edge of the backing to serve as an entry point for the fluid (Figure 6a). The addition of a protein-based analyte happened at different stages during the different experiments. To investigate how a proteinous analyte is transported along the paper strip, three settings were tested (Figure 6b). In the first setting, the so-called “wet out flow”, the protein was placed on the dry paper before the lateral flow experiment is started. The protein was, however, not allowed to dry completely on the paper, as fully dried protein is almost completely retained on the deposition spot, possibly due to denaturing of the proteins, which can render them insoluble. The retaining of dried protein has been shown in reference experiments, as well as on pure PDMAA networks attached to planar solid substrates (Figure S4: Water uptake in a vertical flow setup with and without heating. In the heated setup a relative increase in water-uptake at equilibrium of about ~75% can be observed [47]). Therefore, in such settings, we did not let the protein solution dry to a complete extent, but started the flow right after deposition of the protein. In the second setting, the so-called “with the flow” experiments, the protein is applied “with the fluid front”. Finally, in the third setting, the so-called “fully wetted flow”, the protein is added significantly behind the propagating fluid front.

Investigations with the described three settings were carried out by using the enhanced green fluorescent protein (EGFP) or fluorescently labelled protein (FITC-BSA), respectively, at different times within the experiment while the position of the protein placement was not changed. After completion of the tests, the strips were analyzed for fluorescence intensity across the length of the strip using a fluorescence imager (Vilber FUSION FX7 EDGE).

In order to quantify the effect of the polymer coatings, the retarding front values (*R_f_* values) were used as a means to describe the average distance a protein covered within the experiment. The *R_f_* value was calculated from the average protein position:

(1) $$\mathrm{Average}\mathrm{protein}\mathrm{position}x_{Prot}=\frac{\sum^{\;}\left(gray\;value\times position\right)}{\sum^{\;}\left(gray\;value\right)}$$

The average protein position can then be used to calculate the *R_f_* value of the protein. The *R_f_* value is the ratio of the distance the protein traveled and the distance the fluid has traveled.

(2) $$R_{f}\mathrm{value}R_{f}=\frac{d_{prot}}{d_{fluid}}$$

The distance of the protein can be described as:

(3) $$d_{Prot}=x_{prot}-1\;\mathrm{cm}$$

This is because the protein is always applied at the 1 cm mark of the strip, which has to be subtracted from the final position. The distance covered by the fluid is slightly different. For the “wet out flow” and “with the flow” experiments, it is 7 cm, as this is the distance where it is in contact with the protein. For the “fully wetted” experiments, it is 5 cm, as the fluid front has already passed the first 3 cm of the strip when the protein is added.

**Figure 7:**
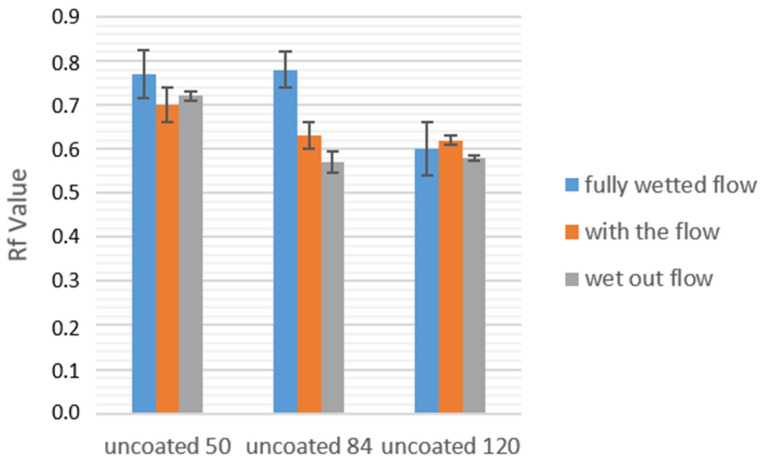
A comparison between cotton linter paper strips of different grammage in terms of the resulting RF values. The applied protein was BSA.

In a first set of flow experiments, we wanted to get more insight into the protein binding behavior during LFTs. To this, we varied the grammage of the paper used and, in addition, the timing at which protein is applied to the paper strip. Varying the grammage affected the imbibition speed without changing the chemical identity of the paper itself, with papers of lower grammage having the faster fluid flow. A general trend visible in this line of experiments is that paper with a lower grammage performs better in terms of protein repulsion. Figure 7 depicts the impact that paper grammage has on the resulting *R_f_* values of the experiments. A reduction for all experimental setups can be seen when comparing the 50 g·m^−2^ with the 120 g·m^−2^ paper. This was visible throughout the experiments, regardless of coating or protein used. This effect can be attributed to an increased fluid transport velocity in papers of lower grammage, which, in turn, results in lower possibilities of interactions of the proteins with the surrounding surface of the paper fibers once being transported in the macropores of the paper sheet.

**Figure 8:**
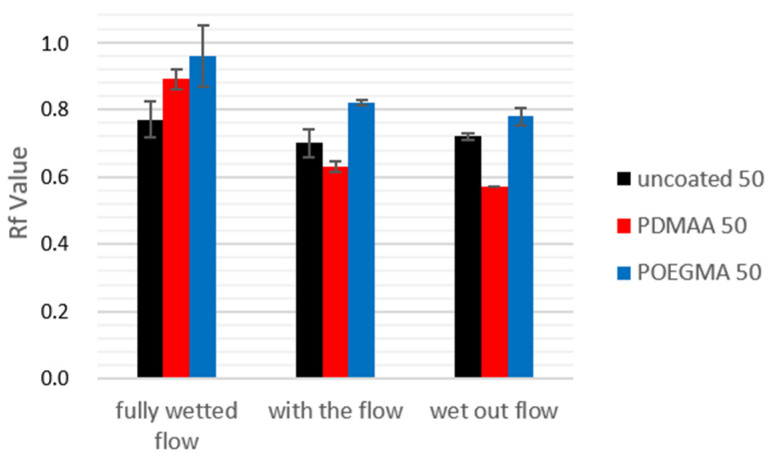
A comparison of different application times within the flow experiment shows a clear advantage of “fully wetted” experiments, no matter the coating.

Next the different polymeric coatings were compared at one given grammage of the used paper sheet. Figure 8 shows the summary of nine experiments using the same grammage paper (50 g·m^−2^), but different coatings and application times. The results show that the POEGMA coating was able to increase the *R_f_* value for all experiments when compared to uncoated paper. Occasional *R_f_* values of more than 1.0 were achieved, which can be attributed to an apparent reduction in accessible volumes for molecules being transported in the sheet due to, e.g., size exclusion effects of the hydrogel coating and the swollen paper fiber or electrostatic repulsion between the negatively charged fiber surface and molecules of similar charge. The latter has been recently studied in detail and the reader is referred to the literature for details [48]. The PDMAA-based coating was also able to reduce protein retention in the case of a “fully wetted flow”, but performed worse than uncoated paper for the “wet out flow” and “with the flow” experiments. In experiments where higher grammage paper was used, PDMAA always resulted in a decrease in the *R_f_* value when compared to uncoated paper, indicating a higher loss of analyte across the paper. A proposed reason for the counterintuitive effects of the PDMAA coating is the reduction in fluid imbibition and speed in PDMAA-coated paper strips, which itself is a direct result of a higher contact angle between the fiber surface and water. As seen in the data in Figure 7, fluid transport velocity can directly influence the retention of proteins on the paper fibers. For a strip to become fully soaked in our horizontal assay, uncoated paper, on average, took 4:30 min, while, for PDMAA-coated paper, this time increased to around 10 min. In contrast, POEGMA-coated paper even turned out faster than uncoated paper, with a flow time of 3:30 min. Coupled with the increased diffusion at longer flow times, the higher chance for a protein to adsorb at lower fluid speeds leads to an accumulation of proteins earlier on in the paper strip, despite the reduction in protein binding to PDMAA-coated paper in the static experiments shown above in Figure 5. In a further comparison of the three application methods, we can finally observe the similarity in *R_f_* values of the three uncoated papers. Once a coating is applied to the paper fibers, the application time does make difference. The data in Figure 8 shows that under “fully wetted flow” conditions, larger *R_f_* values can be achieved; hence, a reduced interaction between fibers and protein is observed. Both “with the flow” and “wet out flow” experiments exhibit lower *R_f_* values, which is most likely due to the fact that the protein is deposited onto a hydrogel in its non-swollen state. As the impact of the hydrogel is largest when the gel is fully swollen, the fully wetted flow delivers the lowest protein retention.

**Figure 9:**
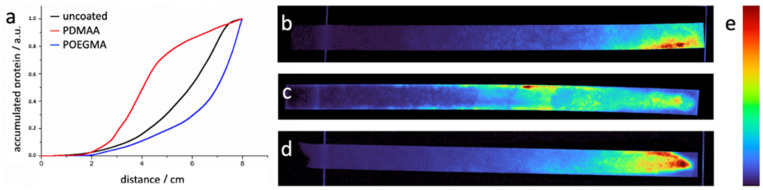
(**a**) A comparison of three experiments with the same conditions, but differently coated paper. The protein GFP was applied “with the flow” after 1 cm on the paper strip. The fluid used is PBS and the experiment is stopped once the entire paper is wetted. (**b**) The POEGMA coating allows the protein to reach the end of the strip and little protein is visible at the beginning and center of the strip. (**c**) The PDMAA-coated paper shows high retention of proteins. (**d**) The uncoated paper shows that the protein does not reach the end of the paper strip and has a lot of residue across the strip. (**e**) The look-up table stretches from violet (low signal intensity) to red (high signal intensity).

Even though the *R_f_* values give a good first orientation about the retention of the proteins along the paper strip, we were interested in further quantifying the exact protein distribution by fluorescence imaging. The latter was done by fitting the gray value distribution across the length and width of the paper strip, which allowed us to image and highlight regions where protein was more concentrated. By normalization and changing the gray value data to cumulative data, we can see different slopes emerge that can be compared across experiments. In Figure 9, three graphs are overlaid, each showing the accumulation of protein signal across the length of a paper strip, either coated with POEGMA (blue) or PDMAA (red), respectively, or uncoated (black). The graph shows that POEGMA-coated paper shows most of its protein signal towards the end of the paper strip (more than 70% after the 6 cm mark), while uncoated paper (~50%) and PDMAA-coated paper (<20%) exhibit more fluorophores at earlier parts of the strip. The representing test strips are shown on the right side of the figure as a reference.

### 3.5. Protein Accumulation by Increased Flow Volume via Localized Heating

**Figure 10:**
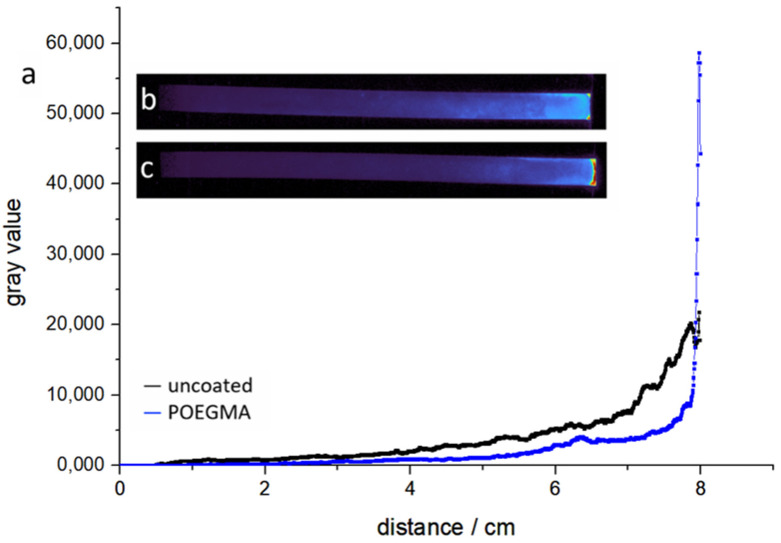
A comparison of uncoated and POEGMA-coated paper. In this experiment, BSA-FITC is in a “fully wetted flow” experiment setup. The fluid was evaporated at 7.5–8 cm of the strip for 30 min once the strip was fully soaked. (**a**) The protein position across the strip by the means of gray values; (**b**) the fluorescent image of uncoated cotton linters. (**c**) The fluorescent image of POEGMA-coated cotton linters.

Following the observation that P(OEGMA-co-MABP) functionalization is able to significantly reduce protein retention, we aimed to extend the duration of liquid flow through the paper strip in order to see how much of the applied protein could be transported without becoming retained. For this, we applied heat at the end of the strip (40 °C) in order to cause evaporation and, consequently, an extended flow of fluid. Reference experiments show that, by evaporating at the last 10% of the paper strip, an increase in fluid uptake of roughly 85% can be achieved in the equilibrium state. Evaporating for 30 min increases the total volume of water that flows through the paper by approximately 40%. The heating setup is depicted in Figure S3 and the results of the reference experiment in Figure S4. In Figure 10, we used the “fully wetted flow” configuration, as it ensures that the hydrogel is swollen before the protein is applied. Similar to the experiments without heating, the strips were connected to the fluid reserve, and a timer was started once the paper was fully soaked. After 30 min, the paper was disconnected from the fluid and analyzed, as previously. The higher concentration of fluorophores towards the end of the strip only required some adjustments to the exposure time during image acquisition in order to avoid saturation of the signal.

**Figure 11:**
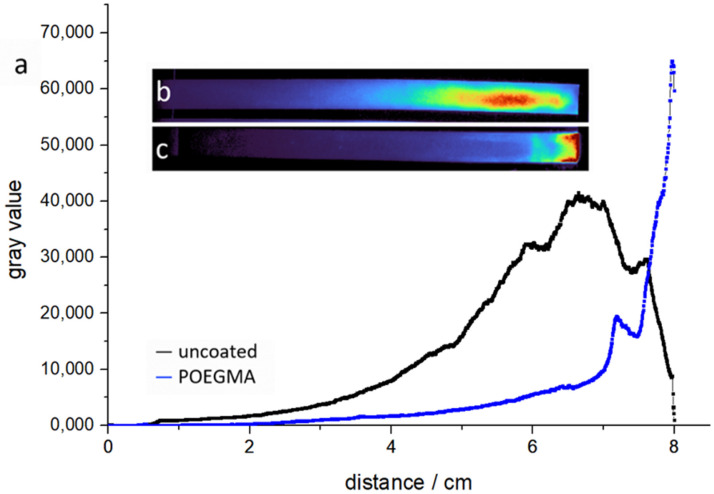
A comparison of uncoated and POEGMA-coated paper. In this experiment, GFP with a surface charge of +9 is applied in a “fully wetted flow” setup. The fluid was evaporated at 7.5–8 cm of the strip for 30 min once the strip was fully soaked. (**a**) The protein position across the strip by the means of gray values; (**b**) the fluorescent image of uncoated cotton linters. (**c**) The fluorescent image of POEGMA-coated cotton linters.

The results clearly show that the majority of protein remains mobile and can be transported throughout the paper strip under conditions of extended fluid flow, regardless of whether POEGMA-coated or uncoated paper is being used. The reason for this may be attributed to weak binding of the negatively charge dissolved BSA or EGFP to the negatively charged stationary coated or uncoated fiber. In order to prove this hypothesis, we further studied a positively charged protein and its retention in the coated/uncoated paper strips. As a model, we applied a positively charged version of GFP in a similar fashion as before for the negatively charged proteins. We again analyzed the retention by means of fluorescent imaging, and Figure 11 shows comparative images and cross-section analysis of the GFP+9 retention on paper and POEGMA-coated paper, respectively. This allows us to compare the same protein (almost identical fold) with a different charge to its negatively charged native counterpart [49].

A fully wetted flow under thermal treatment in the final 0.5 cm zone now results in a different behavior for an uncoated paper sheet. The GFP+9 protein is strongly retained; even with solvent evaporation at the end-zone, it cannot be concentrated in this zone. Other than that, the same protein behaves differently in POEGMA-coated paper. Here, similarly, the negatively charged GFP analogue, the GFP+9, can be transported all the way to the end-zone during thermal evaporation. The latter suggests that electrostatics can also play an important role and may not be neglected when designing a specific lateral flow test with such polymer-modified paper sheets.

## 4. Discussion

Protein adsorption on paper-based analytical devices can be reduced by coating cellulose fibers with polymer hydrogels, as demonstrated here, in binding studies using a highly cellulose affine protein CBM3a–mRuby3 and CLSM image analysis. However, the extent to which a reduction was obtained in lateral flow experiments varied between the hydrogels.

In the case of the PDMAA-based coating, the hydrogel coating increased the contact angle of water with the cellulose surface, changing the capillary pressure and, thereby, the imbibition times significantly. The resulting slower flow rates allowed for higher probability of interactions between fibers and the proteins, increasing the amount of protein retained on the paper fibers. The slower flow rate not only increased the protein retention, but also reduced the distance of GFP and BSA-FITC across the paper strip. We attributed this effect to the reduced flow that is strongly influenced by the coating itself.

In the case of POEGMA-based copolymer coating, the contact angle of P(OEGMA-co-MABP) on a planar surface yielded a contact angle of roughly 30° after one minute, which is comparable to values from pure cellulose model surfaces [50]. The POEGMA coating reduced the amount of retained proteins at the early stages of the paper strips and concentrated them towards the end. This effect is attributed to the previously described “entropic shielding” effect. It entails that proteins, or generally macromolecules of a certain size, will not be able to penetrate into a swollen and crosslinked hydrogel network due to the significant loss in entropy that a further stretching of the hydrogel induced by the penetrating molecules would involve [41]. A similar effect might be caused by size exclusion when the protein molecules are larger than the mesh size of hydrogel. It stands to reason that the entropic shielding effect of a hydrogel is generally useful to reduce protein adsorption on fibers. When working in a lateral flow setup, however, as is shown in the comparison of the different hydrogel coatings, the fluid transport rates also play a major part in the retention of proteins, putting more demands on the coating than previously thought.

Despite the fact that the two proteins used in this study had a negative surface charge, like the cellulose, POEGMA reduced the protein binding when negatively charged proteins were used in model flow assays. If positive proteins are used, the improvement by the POEGMA coating relative to uncoated paper is even more pronounced, as positively charged proteins are quite strongly retained by the negatively charged cellulose surface of the paper fibers. Note, the negative charge stems from native oxidation processes typically taking place in cellulose pulp at the C6 position of the glucose units and is practically an intrinsic component. Especially when biological solutions containing complex protein mixtures are analyzed, there will be always some proteins with positive (partial) charges present.

In the final experiments, using localized heating at an end-zone of the paper strip, where the evaporation induces a pull of the liquid and induces a further increase in the fluid transport through the paper strip, is beneficial to reduce protein binding.

It should be noted that not only the coating, but also the type of paper itself had a strong influence on the retention of the polymer. Paper with a basis weight of 120 g·m^−2^ showed much lower *R_f_* values than their 84 or 50 g·m^−2^ counterparts. This effect was linked to the slower fluid flow that can be attributed to differences in the capillary forces of the respective paper sheets due to different median pore sizes. Furthermore, we were able to see that an application of the protein using a fully wetted flow setting also results in an increase in the *R_f_* value. This effect is attributed to two reasons. The first being the fact that the hydrogel coating is in a swollen state before the protein came into contact with the surface. This effect is especially visible for some of the PDMAA experiments, where “with the front” and “wet out” experiments showed much worse results.

## 5. Conclusions

Hydrogel modification of paper samples via photo-induced crosslinking is able to significantly reduce protein binding to the fibers through entropic shielding and/or size exclusion effects. However, when choosing the hydrogel, one has to pay attention that electrostatic interactions can also play a crucial role with respect to the retention of proteins on the fiber. Neutral hydrogel coatings are particularly important when proteins with a net positive charge are studied, as they adhere particularly strongly on unmodified paper [48].

However, the experiments also show very clearly that this is not a one-dimensional problem. The coating will also change the wetting properties of the paper. Especially when the hydrogel coating is dry, the contact angle can be slightly higher than that of the cellulose fibers, which leads to a reduced imbibition speed due to lower capillary forces. Flow experiments indicate that a reduced imbibition speed and accordingly lower flow rate increases the retention of proteinous analyte across the paper strip. A similar effect is obtained if the flow rate is influenced by intrinsic paper parameters, such as pore size being varied, e.g., by control of the paper grammage. Hence, our experiments demonstrate that low protein binding with microfluidic paper is a result of the interplay of defined surface chemistry (i.e., fiber coatings) and controlled flow rates, where the latter can be adjusted by paper-intrinsic parameters (i.e., porosity), as well as by the fiber coat itself.

Finally, a simple small solvent evaporation zone on a paper strip, e.g., at the end of the strip, can be used to accumulate proteins in a distinct zone, which may also be further engineered to increase the S/N value in paper-based flow tests, bringing us closer to an environmentally friendly alternative for lateral flow assays. In addition, future studies will focus more on the fiber-intrinsic parameters that increase or decrease unspecific adsorption of proteins by observing the protein retention of different fiber types and fiber pretreatments, such as beating. In future studies, we will also focus on benchmark tests using paper-only lateral flow assays in order to investigate the impact of our coating in situations where the amount of analyte is even lower than the current detection limit of uncoated paper assays.

## Data Availability

Not applicable.

## Supplementary Materials

The following are available online at https://www.mdpi.com/article/10.3390/s21196348/s1, Figure S1: 1H-NMR of PDMAA-co-MABP, Figure S2: 1H-NMR of POEGMA-co-MABP, Figure S3: The flow setup for evaporation experiments. The back of the paper strip is laying on top of a heating pad that evaporates water at 40 °C, Figure S4: Water uptake in a vertical flow setup with and without heating. In the heated setup an equilibrium uptake of ~75% more water can be observed, Figure S5: Reference experiments from Rühe et al. The figure shows the retained fluorescent signal of a model protein that was dried on a PDMAA hydrogel layer and was subsequently extracted with PBS [47], Table S1: Water uptake of unrefined cotton linters.
