# Supplementary material for: Reducing Unspecific Protein Adsorption in Microfluidic Papers Using Fiber-Attached Polymer Hydrogels
Source: Sensors (Basel). 2021 Sep 23;21(19):6348. doi: 10.3390/s21196348 (PMC8512548; doi:10.3390/s21196348)
Supplement: Supplementary file 1 [file sensors-21-06348-s001.zip › sensors-1382582-supplementary.pdf]

sensors

Supporting Information

Reducing Unspecific Protein Adsorption in Microfluidic Papers using Fiber-Attached Polymer Hydrogels

Alexander Ritter von Stockert [1], Anna Luongo [2], Markus Langhans [1], Thomas Brandstetter [2], Jürgen Rühe [2,*], Tobias Meckel [1], and Markus Biesalski [1,*]

[1] Department of Macromolecular Chemistry and Paper Chemistry (MAP), Ernst-Berl Institute for Chemical Engineering and Macromolecular Chemistry
Technische Universität Darmstadt, 64287, Darmstadt, Germany; alexander.von_stockert@tu-darmstadt.de (A.R.v.S.); markus.langhans@tu-darmstadt.de (M.L.); tobias.meckel@tu-darmstadt.de (T.M.)

[2] Laboratory of Chemistry and Physics of Interfaces, Institute for Microsystems Technology, Technical Faculty, University of Freiburg, 79110, Freiburg, Germany; anna.luongo@imtek.uni-freiburg.de (A.L.);thomas.brandstetter@imtek.uni-freiburg.de (T.B.)

* Correspondence: ruehe@imtek.de (J.R.); biesalski@tu-darmstadt.de or markus.biesalski@tu-darmstadt.de (M.B.)

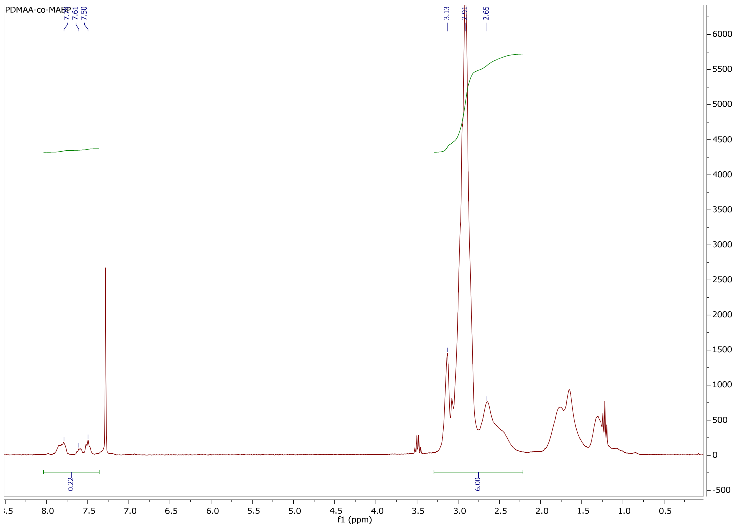

Figure S1: ^{1}H-NMR of PDMAA-co-MABP.

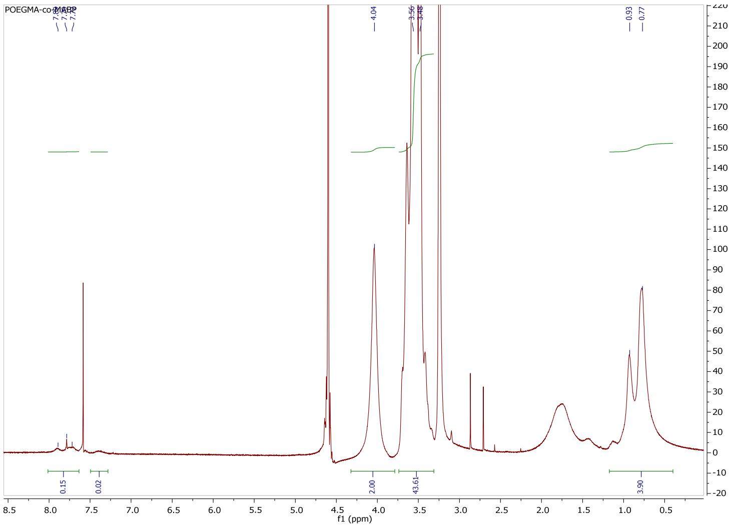

Figure S2: ^{1}H-NMR of POEGMA-co-MABP.

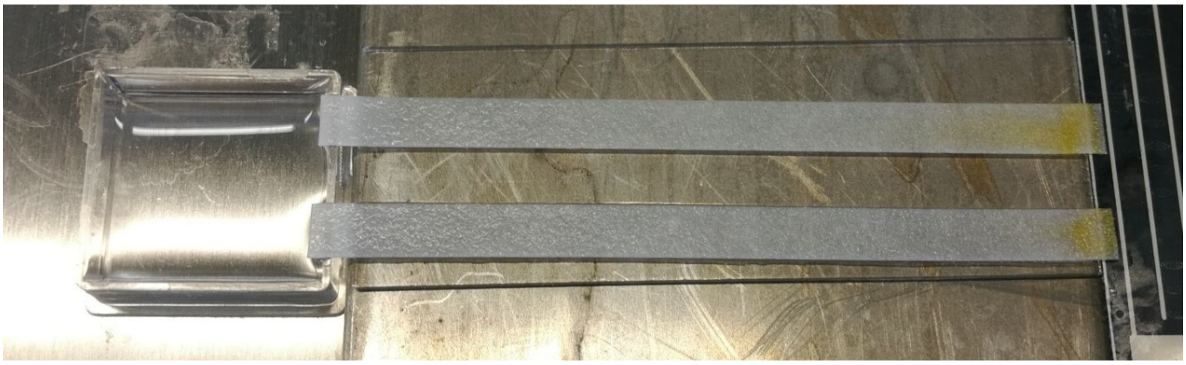

Figure S3: The flow setup for evaporation experiments. The back of the paper strip is laying on top of a heating pad that evaporates water at 40 °C.

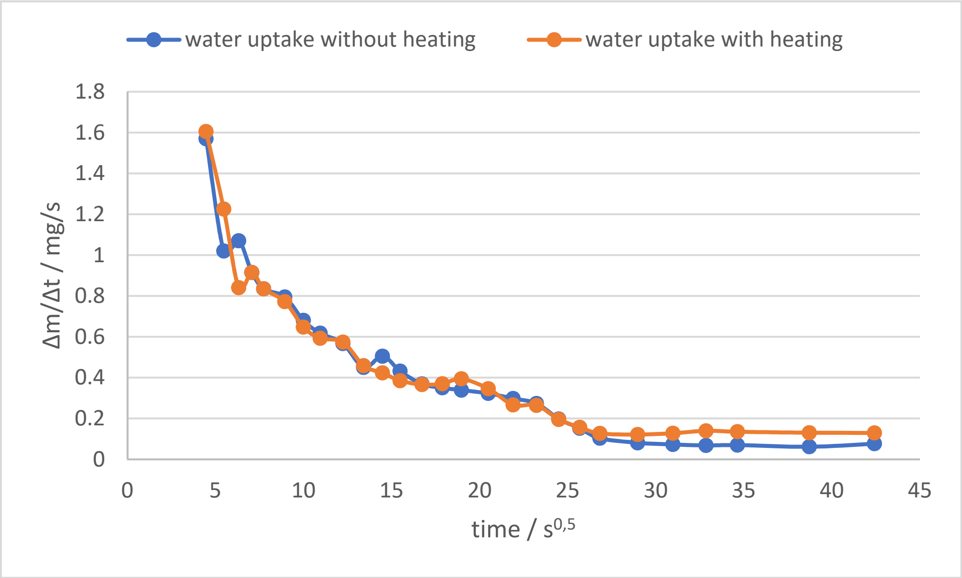

Figure S4: Water uptake in a vertical flow setup with and without heating. In the heated setup an equilibrium uptake of ~75% more water can be observed.

Table S1:Water uptake of unrefined cotton linters

Weight /gm^{-2}	Dry weight / g	Wet weight /g	Difference /g	Weight uptake /%	Avg. uptake /%
50	0.0531	0.3091	0.256	582.11	571.255115
	0.0399	0.2236	0.1837	560.40	
84	0.047	0.2469	0.1999	525.32	528.996208
	0.0505	0.269	0.2185	532.67	

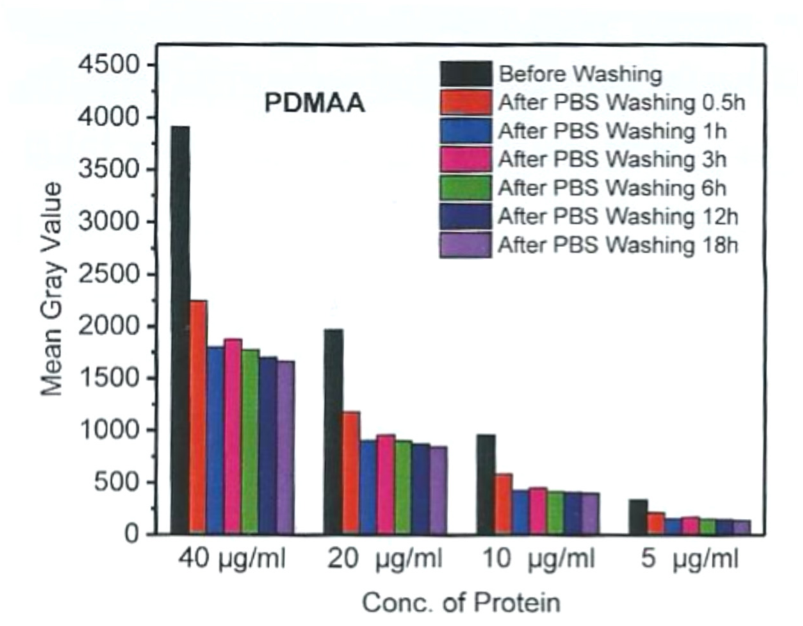

Figure S5: Reference experiments from Rühe et al. The figure shows the retained fluorescent signal of a model protein that was dried on a PDMAA hydrogel layer and was subsequently extracted with PBS. [32]
